# Serological Biomarkers at Hospital Admission and Hospitalization Treatments Are Not Related to Sensitization-Associated Symptoms in Patients with Post-COVID Pain

**DOI:** 10.3390/pathogens12101235

**Published:** 2023-10-12

**Authors:** César Fernández-de-las-Peñas, Carlos Guijarro, Juan Torres-Macho, Oscar J. Pellicer-Valero, Ana Franco-Moreno, Jo Nijs, María Velasco-Arribas

**Affiliations:** 1 Department of Physical Therapy, Occupational Therapy, Physical Medicine and Rehabilitation, Universidad Rey Juan Carlos (URJC), 28922 Madrid, Spain; 2 Department of Internal Medicine-Infectious Department, Research Department, Hospital Universitario Fundación Alcorcón, 28922 Madrid, Spain; carlos.guijarro@urjc.es (C.G.); maria.velasco.arribas@urjc.es (M.V.-A.); 3 Department of Medicine, Universidad Rey Juan Carlos (URJC), 28922 Madrid, Spain; 4 Department of Internal Medicine, Hospital Universitario Infanta Leonor-Virgen de la Torre, 28031 Madrid, Spain; juan.torresm@salud.madrid.org (J.T.-M.); anaisabel.franco@salud.madrid.org (A.F.-M.); 5 Department of Medicine, School of Medicine, Universidad Complutense de Madrid, 28040 Madrid, Spain; 6 Image Processing Laboratory (IPL), Universitat de València, Parc Científic, 46980 València, Spain; oscar.pellicer@uv.es; 7 Pain in Motion Research Group (PAIN), Department of Physiotherapy, Human Physiology and Anatomy, Faculty of Physical Education & Physiotherapy, Vrije Universiteit Brussel, 1050 Ixelles, Belgium; jo.nijs@vub.be; 8 Chronic Pain Rehabilitation Center, Department of Physical Medicine and Physiotherapy, University Hospital Brussels, 1050 Ixelles, Belgium; 9 Department of Health and Rehabilitation, Unit of Physiotherapy, Institute of Neuroscience and Physiology, Sahlgrenska Academy, University of Gothenburg, 41390 Göterbog, Sweden

**Keywords:** COVID-19, sensitization, post-COVID, biomarkers, hospitalization

## Abstract

Current evidence suggests that a group of patients who had survived coronavirus disease, 2019 (COVID-19) and developed post-COVID pain can exhibit altered nociceptive processing. The role of serological biomarkers and hospitalization treatments in post-COVID pain is unclear. This study aimed to investigate the association of serological biomarkers and treatments received during hospitalization with sensitization-associated symptoms in COVID-19 survivors with post-COVID pain. One hundred and eighty-three (n = 183) patients who had been hospitalized due to COVID-19 in one urban hospital of Madrid (Spain) during the first wave of the pandemic were assessed in a face-to-face interview 9.4 (SD 3.4) months after hospitalization. Levels of 19 serological biomarkers, hospitalization data, and treatments during hospitalization were obtained from hospital records. Sensitization-associated symptoms (Central Sensitization Inventory, CSI), sleep quality (Pittsburgh Sleep Quality Index, PSQI), pain catastrophism (Pain Catastrophizing Scale), and anxiety/depressive level (Hospital Anxiety and Depression Scale, HADS) were assessed. The prevalence of post-COVID pain was 40.9% (n = 75). Twenty-nine (38.6%) patients had sensitization-associated symptoms. Overall, no differences in hospitalization data and serological biomarkers were identified according to the presence of sensitization-associated symptoms. The analysis revealed that patients with sensitization-associated symptoms exhibited higher lymphocyte count and lower urea levels than those without sensitization-associated symptoms, but differences were small. Pain catastrophism and depressive levels, but not fatigue, dyspnea, brain fog, anxiety levels, or poor sleep, were higher in individuals with sensitization-associated symptoms. In conclusion, this study revealed that sensitization-associated post-COVID pain symptoms are not associated with serological biomarkers at hospital admission and hospitalization treatments received.

## 1. Introduction

Current evidence supports that Severe Acute Respiratory Syndrome Coronavirus 2 (SARS-CoV-2), the virus responsible of the coronavirus disease 2019 (COVID-19), can provoke symptoms once the acute infection has surpassed. The presence of symptoms at a post-acute phase has received different terms, with long-COVID or post-COVID-19 condition being the most used. The term long-COVID is proposed for describing the presence of any post-COVID symptom after surpassing a SARS-CoV-2 infection [1]. The term post-COVID-19 condition is defined as “a condition occurring in people with a history of probable or confirmed SARS-CoV-2 acute infection, usually three months from the onset of COVID-19 with symptoms that last for at least two months after and cannot be explained by an alternative medical diagnosis” [2]. 

More than 100 post-COVID symptoms have been described [3]. In the last years, several meta-analyses have found that up to 50% of COVID-19 survivors can develop any post-COVID symptom lasting from months [4,5] up to one [6,7] or two [8] years after infection. Pain is a post-COVID symptom experienced by 15–20% of subjects after COVID-19 [9]. This meta-analysis reported pooled prevalence rates ranging from 5% to 20% for different post-COVID pain syndromes, e.g., myalgia, joint pain, and chest pain the six months after the infection [9]. A recent meta-analysis reported similar prevalence rates one-year after the initial infection [10]. However, it is notable to remark that studies included in these meta-analyses have investigated post-COVID pain integrated in the overall post-COVID-19 condition [9,10]. Interestingly, studies focusing on post-COVID pain have reported higher prevalence rates (40–60%) [11,12,13,14]. Thus, pain could be an underestimated post-COVID symptom.

In addition, some studies have tried phenotyping post-COVID pain. Evidence suggests that post-COVID pain primarily resembles musculoskeletal phenotypic features [15]. Musculoskeletal pain is associated with altered nociceptive processing or sensitization [16], which is the underlying mechanism proposed for nociplastic pain [17]. Nociplastic pain is associated with exaggerated responses as well as central nervous system-derived symptoms, e.g., fatigue, sleep problems, memory loss, and mood disturbances [18]. Some studies have identified the presence of sensitization-associated symptoms, assessed by Central Sensitization Inventory (CSI), in a range between 30% [19] and 70% [20] of patients with post-COVID pain. A recent pilot study identified the presence of a deficient conditioned pain modulation (CPM) in almost 15% of patients with post-COVID pain [21]. Identification of risk factors associated with sensitization-associated symptoms in individuals with post-COVID pain could help more personalized interventions. Evidence supports a close interaction between chronic pain, sensitization, and altered immune system functioning [22]. Thus, the immune response associated with SARS-CoV-2 infection may promote immune factors, potentially contributing to develop or promote pain sensitization, especially if they cross the blood–brain barrier [23]. 

Different hematological (lymphocyte count, neutrophil count), inflammatory (C-reactive protein [CRP]), immunological (interleukin IL-6) or biochemical (creatine kinase [CK], D-dimer) biomarkers have been investigated at the acute phase of SARS-CoV-2 infection to identify subjects at a higher risk of severe COVID-19. For instance, patients with severe COVID-19 have higher CRP, D-dimer, and lactate dehydrogenase (LDH) levels but lower albumin levels than those with non-severe COVID-19 [24]. The role of these serological biomarkers in post-COVID is less understood and current evidence is highly heterogeneous [25]. A recent meta-analysis pooling data from 24 biomarkers found that individuals with post-COVID symptoms exhibited higher levels of CRP, D-dimer, LDH, and leukocytes when compared with survivors without post-COVID symptomatology [26]. However, this meta-analysis pointed out that throughout sensitivity analyses by groups of long-haulers, these biomarker changes can be symptom-specific as well as chronicity specific [26]. Furthermore, it is important to consider if the serological biomarkers were obtained at the acute phase of the infection or at the time of assessment of post-COVID symptoms [26]. 

The specific association between serological biomarkers and post-COVID pain is also heterogeneous. Bakılan et al. observed lower lymphocyte count but higher D-dimer levels at hospital admission in patients developing musculoskeletal post-COVID pain [27]. Fernández-de-las-Peñas et al. reported that patients with post-COVID pain showed a higher lymphocyte count but lower glucose and creatine kinase levels at hospital admission than those without post-COVID pain eight months after the infection [28]. These discrepancies have been identified in a recent meta-analysis [10].

Monitoring serological biomarkers at the acute phase of the infection could help for early identification of subjects at a higher risk of developing post-COVID pain. Thus, different serological biomarkers could be associated with specific post-COVID symptoms. A previous study found that COVID-19 survivors with sensitization-associated symptoms showed lower ferritin and hemoglobin levels at hospital admission than those without sensitization-associated symptoms, but the differences were small [29]. This study included a small sample, just patients with post-COVID pain, and did not control for hospitalization treatments. In fact, no previous study has controlled hospitalized treatments in relation to the development of post-COVID pain. Since pain may also be related to hospitalization treatments, and particularly medications that could affect the nociceptive system and promote chronic pain [30], further studies are needed. 

This study primarily aimed to investigate the association of serological biomarkers at hospital admission and treatments received during hospitalization with the development of sensitization-associated symptoms in individuals who had been previously hospitalized due to COVID-19 and develop post-COVID pain. In addition, a secondary aim was to identify if individuals who develop sensitization-associated symptomatology also exhibit other central nervous system-derived symptoms such as fatigue, sleep problems, brain fog, and mood disturbances.

## 2. Methods

### 2.1. Participants

Patients who had been hospitalized due to SARS-CoV-2 acute infection between March and May 2020 (first wave of the pandemic) at an urban hospital in Spain were invited to participate in the study. To be included, the diagnosis of SARS-CoV-2 infection was confirmed by a real-time reverse transcription-polymerase chain reaction (PCR) assay of a nasopharyngeal and/or oral swab sample. Thus, patients should also report clinical and radiological findings at hospital admission. The local ethics committee of the hospital (HUFA20/126) approved the study. All participants provided their informed consent at the time of the interview. The study was conducted following the strengthening the reporting of observational studies in epidemiology (STROBE) guideline [31], which is shown as a Supplementary File.

### 2.2. Hospitalization Data 

Clinical and hospitalization data including onset symptoms of COVID-19 at hospital admission, pre-existing medical comorbidities, oxygen therapy, and days at hospital were systematically collected at hospitalization. Blood samples were routinely extracted from all hospitalized patients due to SARS-CoV-2 infection at admission. Thus, serological levels of the following biomarkers were systematically collected: C-Reactive Protein (CRP), lactate dehydrogenase (LDH), creatine kinase (CK), lymphocyte count, neutrophil count, lymphocyte count, alanine transaminase (ALT), aspartate transaminase (AST), bilirubin, glucose, hematocrit, platelet count, D-dimer, albumin, urea, sodium, and potassium, as well as activated partial thromboplastin time (APTT).

Further, treatments received during hospitalization were also registered from the following list: oral corticoids, inhaled corticoids, statins, antiaggregant, anticoagulant, non-steroidal anti-inflammatory drugs (NSAIDs), heparin, antibiotics, Tocilizumab, Lopinavir/Ritonavir, Hydroxychloroquine, Methylprednisone, or Dexamethasone.

### 2.3. Collection Data

Participants were scheduled for a personal face-to-face appointment conducted by trained healthcare researchers. Participants were asked for the presence of pain starting the following four months after hospitalization and whether the pain persisted at the time of the study. We defined post-COVID pain as symptoms compatible with a diagnosis of chronic primary musculoskeletal pain, as defined by the International Association for the Study of Pain (IASP) [32], experienced for at least three consecutive months after hospital discharge, and in the absence of any underlying medical condition which could best explain pain, e.g., arthritis. We excluded headaches due to the need for a diagnosis according to agreed classifications. We also asked for the self-presence of fatigue, dyspnea, and brain fog since these post-COVID symptoms are highly comorbid with pain. In patients with pain symptoms, a 11-point numerical pain rating scale (NPRS, 0–10) was used to assess its intensity.

A structured questionnaire including several patient-reported outcome measures (PROMs) evaluating sensitization-associated symptoms (Central Sensitization Inventory, CSI) [33], anxiety and depressive levels (Hospital Anxiety and Depression Scale, HADS) [34], sleep quality (Pittsburgh Sleep Quality Index, PSQI) [35], and pain catastrophism (Pain Catastrophizing Scale, PCS) [36] was used. 

The CSI evaluates 25 associated symptoms assumed to represent aspects of central sensitization based on 5-point Likert scale rating [33]. The total score ranges from 0 to 100 points, where a cut-off ≥40 points suggests the presence of sensitization-associated symptomatology [37]. The CSI score has good psychometric properties for assessing sensitization-associated symptoms in individuals with chronic pain [38].

The HADS consists of 7 items assessing anxiety (HADS-A, 21 points) and 7 items evaluating depressive (HADS-D, 21 points) symptomatology [34]. A cut-off score of ≥8 points on each subscale has shown good sensitivity and specificity for identifying anxiety or depressive symptomatology [39]. The HADS has shown good validity in people with long-COVID [40]. 

The PSQI evaluates sleep quality the previous month by including 19 self-rated questions about usual bed-time, usual wake time, number of hours slept, and number of minutes to fall asleep which are answered on a 4-point Likert-type scale (0–3). The total score ranges from 0 to 21 points, where a cut-off score ≥ 8 points is indicative of poor sleep quality [35]. The PSQI has shown good internal consistency and reliability [41]. 

The PCS consists of 13 items assessing rumination, magnification, and despair aspects in relation to the subject’s pain experience. The responses are rated from 0 (never) to 4 (always), leading to a score ranging from 0 to 52 points [36].

### 2.4. Statistical Analysis

Data analysis was conducted with STATA 16.1 program (StataCorp. 2019. Stata Statistical Software: Release 16. College Station, TX: StataCorp LP. TX, USA). Missing values were imputed by using median imputation. The Shapiro–Wilk test was used to assess the assumption of normality. First, McNemar’s Chi-squared test and paired Student *t*-tests were used to compare proportions and means between patients with and without post-COVID pain (without considering the presence or not of sensitization-associated symptoms). Second, a one-way ANOVA was used to compare all variables between patients without post-COVID pain, patients with post-COVID pain and CSI < 40 points, and patients with post-COVID pain and CSI ≥ 40 points. For all inferences, a corrected *p*-value of < 0.05 was considered significant.

## 3. Results

### 3.1. Participants

One hundred and eighty-three patients (n = 183) from an initial sample of 220 patients were included in this study. Twenty patients refused to participate and the remaining 17 were excluded because blood samples did not include all biomarkers. Participants were assessed a mean of 9.4 ± 3.4 months after hospitalization. The prevalence of post-COVID pain symptoms in the sample was 40.9% (n = 75).

### 3.2. Post-COVID Pain 

No overall significant differences in demographic data (sex: *p* = 0.742; age: *p* = 0.508; weight: *p* = 0.862; height: *p* = 0.903), previous medical co-morbidities (number: *p* = 0.534; hypertension: *p* = 0.754; obesity: *p* = 0.240; cardiovascular diseases: *p* = 0.688; asthma: *p* = 0.475; chronic obstructive pulmonary disease: *p* = 0.340), pre-infection pain symptoms (*p* = 0.988), onset symptoms of COVID-19 at hospital admission (number: *p* = 0.612; fever: *p* = 0.708; dyspnea: *p* = 0.871; myalgias: *p* = 0.635; headache: *p* = 0.533; cough: *p* = 0.599; diarrhea: *p* = 0.748; anosmia: *p* = 0.730; ageusia: *p* = 0.261; throat pain: *p* = 0.245; vomiting: *p* = 0.316; dizziness: *p* = 0.224) and hospital stay (*p* = 0.401) between patients with and without post-COVID pain were identified (Table 1). The only significant difference was that a greater proportion of patients with pre-infection diabetes developed post-COVID pain (*p* = 0.02).

No differences in treatment received at the hospital during the acute phase were either observed according to the presence or absence of post-COVID pain (Table 1): oral corticoids (*p* = 0.493), inhaled corticoids (*p* = 0.129), antiaggregant (*p* = 0.776), heparin (*p* = 0.650), statins (*p* = 0.123), anticoagulants (*p* = 0.509), NSAIDs (*p* = 0.446), antibiotics (*p* = 0.751), oxygen therapy (*p* = 0.201), Hydroxychloroquine (*p* = 0.382), Tocilizumab (*p* = 0.640), Methylprednisone (*p* = 0.822), Dexamethasone (*p* = 0.964), and/or Lopinavir/Ritonavir (*p* = 0.951).

Patients suffering from post-COVID pain showed lower LDH levels (*p* = 0.01) and higher neutrophil count (*p* = 0.04) at hospital admission than those without post-COVID pain (Table 2). No significant differences between patients with and without post-COVID pain for the remaining biomarkers were found: CRP (*p* = 0.361), APTT (*p* = 0.462), leucocyte count (*p* = 0.228), lymphocyte count (*p* = 0.267), CK (*p* = 0.313), ALT (*p* = 0.294), AST (*p* = 0.153), bilirubin (*p* = 0.938), glucose (*p* = 0.447), hematocrit (*p* = 0.21), platelet count (*p* = 0.998), D-dimer (*p* = 0.376), albumin (*p* = 0.280), urea (*p* = 0.576), sodium (*p* = 0.248), and potassium (*p* = 0.858) levels (Table 2).

### 3.3. Sensitization-Associated Symptomatology

From 75 patients with post-COVID pain, 29 (38.6%) exhibited sensitization-associated symptomatology (CSI score 54.9, SD 9.1). No differences in demographic data (sex: *p* = 0.324; age: *p* = 0.798; weight: *p* = 0.974; height: *p* = 0.6408), pre-existing medical co-morbidities (number: *p* = 0.790; hypertension: *p* = 0.943; diabetes: *p* = 0.765; obesity: *p*= 0.496; asthma: *p* = 0.755; cardiovascular disease: *p* = 0.671; chronic obstructive pulmonary disease: *p* = 0.543), pre-existing pain symptomatology (*p* = 0.574), COVID-19 onset symptoms at hospital admission (number: *p* = 0.290; fever: *p* = 0.928; dyspnea: *p* = 0.731; myalgias: *p* = 0.639; cough: *p* = 0.860; headache: *p* = 0.771; diarrhea: *p* = 0.501; anosmia: *p*= 0.935; ageusia: *p* = 0.132; throat pain: *p* = 0.457; vomiting: *p* = 0.564; dizziness: *p* = 0.110), and days at hospital (*p* = 0.256, Table 3).

No significant differences in hospitalization treatment received were either found (Table 3): oral corticoids (*p* = 0.765), inhaled corticoids (*p* = 0.132), heparin (*p* = 0.722), statins (*p* = 0.289), antiaggregant (*p* = 0.929), anticoagulants (*p* = 0.521), NSAIDs (*p* = 0.525), antibiotics (*p* = 0.596), oxygen therapy/no intubation (*p* = 0.309), Hydroxychloroquine (*p*= 0.660), Tocilizumab (*p* = 0.883), Methylprednisone (*p* = 0.830), Lopinavir/Ritonavir (*p*= 0.201), and/or Dexamethasone (*p* = 0.539). 

No overall differences between patients with and without sensitization-associated symptoms for the following serological biomarkers were identified: CRP (*p* = 0.585), APTT (*p* = 0.288), leucocyte count (*p* = 0.466), CK (*p* = 0.232), ALT (*p* = 0.394), AST (*p* = 0.266), bilirubin (*p* = 0.453), glucose (*p* = 0.574), hematocrit (*p* = 0.126), platelet count (*p* = 0.992), D-dimer (*p* = 0.343), albumin (*p* = 0.132), sodium (*p* = 0.478), and potassium (*p* = 0.964) levels. Post hoc analyses in relation to LDH levels and neutrophil count revealed that differences were not significant between patients with and without sensitization-associated symptoms (Table 4). The analysis considering the presence of sensitization-associated symptoms identified that individuals suffering from post-COVID pain and sensitization-associated symptoms had a higher lymphocyte count (*p* = 0.02) and lower urea levels (*p* = 0.03) at hospital admission than those subjects without sensitization-associated symptoms (Table 4).

### 3.4. Other Associated Symptomatology

No differences were identified for the presence of other post-COVID symptoms such as fatigue (*p* = 0.701), brain fog (*p* = 0.392), or dyspnea (*p* = 0.162), as well as anxiety levels (HADS-A score: *p* = 0.285; cut-off score: *p* = 0.7898), poor sleep (PSQI score: 0.124; cut-off score: *p* = 0.121), or pain intensity (*p* = 0.891) between individuals with and without post-COVID pain (with and without sensitization-associated symptoms, Table 5). The analysis observed higher pain catastrophism (PCS: *p* = 0.251), and depressive (HADS-D score: *p* = 0.03; cut-off score: *p* = 0.025) levels in individuals with sensitization-associated symptoms. 

## 4. Discussion

This study found that 38.6% of COVID-19 survivors reporting post-COVID pain had sensitization-associated symptoms. Overall, no differences in hospitalization treatment and serological biomarkers at the acute phase of the infection were identified according to the presence/absence of sensitization-associated symptoms. Post hoc analyses revealed that patients with sensitization-associated symptoms showed higher lymphocyte count and lower urea levels than those without sensitization-associated symptomatology, but the differences were small. Pain catastrophism and depressive levels, but not fatigue, dyspnea, brain fog, anxiety levels, or poor sleep, were also higher in individuals with sensitization-associated symptoms. 

The presence of sensitization-associated symptoms in patients with post-COVID pain has been previously observed [19,20,21]. In fact, it has been previously suggested that some individuals with post-COVID pain can exhibit nociplastic features and may require a more nuanced multimodal treatment approach to achieve better treatment outcomes [42]. This study found that almost 40% of patients with post-COVID pain (n = 29, 15.8% of the sample) exhibited sensitization-associated symptoms as well as central nervous-derived symptoms, e.g., fatigue, brain fog, sleep problems, or psychological disturbances. Thus, these central nervous-derived symptoms were not associated with the presence of post-COVID pain or sensitization-associated symptoms since they were present in all groups of COVID-19 survivors. Our findings agree with the current literature supporting that fatigue, dyspnea, brain fog, or pain are the most prevalent post-COVID symptoms [4,5,6,7,8]. Thus, the presence of pain in a subgroup of COVID-19 survivors could promote the development of sensitization-associated symptoms and altered nociceptive processing. In fact, the presence of sensitization-associated symptoms could be included in surveillance models for screening of individuals with post-COVID symptoms for helping clinicians to identify treatment priorities [43].

### 4.1. Serological Biomarkers at Hospital Admission

Due to the heterogeneity of post-COVID condition, early identification of potential factors associated with a higher risk of developing more complex clinical presentations is needed. In such a scenario, identification of the factors during the acute phase of SARS-CoV-2 infection could help identify those subjects at a higher risk. Hospitalization due to COVID-19 itself represents a factor potentially associated with the development of post-COVID-19 condition, although evidence on this association is conflicting [44]. At hospitalization, serological biomarkers have received increased attention, particularly in their association with severe COVID-19 [24]. However, data on serological biomarkers at the acute phase of the infection and long-COVID is heterogeneous [25,26]. Although some serological biomarkers have been associated with the post-COVID-19 condition [26], due to the complexity and heterogeneity of the condition, this association seems to be symptom-specific. 

Thus, the association between a specific post-COVID symptom, e.g., pain, with serological biomarkers is also heterogeneous [29]. For instance, Bakılan et al. identified a lower lymphocyte count at hospital admission [27] whereas Fernández-de-las-Peñas et al. observed higher lymphocyte count at hospital admission [28] in individuals with post-COVID pain. In the current study we found higher a neutrophil count at hospital admission when comparing patients with and without post-COVID pain. Differences in biomarkers analyzed, follow-up periods, post-COVID clinical features, and heterogeneous underlying mechanisms of post-COVID pain could explain these discrepancies. A previous study observed that the presence of sensitization-associated symptomatology was associated with lower ferritin and hemoglobin levels at hospital admission, but their relevance was small [29]. In the present study, we identified higher lymphocyte count and lower urea levels in individuals with sensitization-associated symptoms, but differences were small. Previous and current evidence would suggest that the presence of sensitization-associated symptoms could be associated with an “exaggerated” immune response (high lymphocyte or neutrophil count) at the acute phase of the infection, but further studies are needed to confirm this assumption.

The rational linking of SARS-CoV-2 and sensitization-associated symptoms would be related to the fact that cytokine and interleukin COVID-19-related storms may lead to sensitization of pain pathways [45,46] by altering the excitatory–inhibitory balance between systems of nociception [47]. We analyzed standardized serological biomarkers commonly used in hospitalization management, but we did not include some specific inflammatory biomarkers associated with cytokines or interleukins at the acute phase of SARS-CoV-2 infection. It is also possible that a long-lasting inflammatory response maintained after the acute phase would lead to central nervous system changes and subsequent nociplastic changes. In fact, the presence of post-COVID symptoms has been associated with higher neutrophil count, neutrophil/leucocyte ratio, fibrinogen, and CRP levels three months after the infection; however, this study remarked that the association between these biomarkers was symptom-specific [48]. Thus, no association between any biomarker and post-COVID pain was either identified in this study [48].

### 4.2. Treatments during Hospitalization

Our study is the first one specifically investigating if treatments received during hospitalization could be associated with the development of post-COVID pain with or without sensitization-associated symptoms. Our study revealed common use of drugs with anti-inflammatory properties, e.g., NSAIDs, corticoids, and antibiotics, in hospitalized patients with SARS-CoV-2 infection. Theoretically, such anti-inflammatory drugs hold potential in preventing the development of central sensitization. Indeed, in inflammatory rheumatic conditions such as osteoarthritis, it is believed that bottom-up peripheral inflammation drives the development of sensitization [16]. However, as the number of patients receiving either of these anti-inflammatory drugs differed between those with and without post-COVID pain (differentiating between those with or without sensitization), our study suggests that inflammation in the acute stage of COVID-19 illness is unlikely to drive the development of long-lasting sensitization in patients who develop post-COVID pain symptomatology. This assumption is also supported by the fact that inflammatory biomarkers were either different between patients with and without sensitization. Still, COVID-19 is able to trigger the development of long-lasting chronic inflammation [49], which, in turn, can lead to central nervous system inflammation [50], for instance via the vagus nerve [51], and subsequent sensitization of nociceptive pain pathways. Future studies should explore this hypothesis. Similarly, the use of some retroviral drugs such as Ritonavir/Lopinavir was not associated with either the development of post-COVID pain nor with sensitization-associated symptomatology. Additionally, preclinical work supports a mild sensitizing effects of statins [52], but the lack of differences in statin use between patients with and without post-COVID-pain refutes an important role for statin use in the development of sensitization-related symptoms following COVID-19 illness. 

### 4.3. Limitations

The results of the current study should be considered according to its potential limitations. First, our cohort only included previously hospitalized COVID-19 survivors, so we do not know if the same results would be observed in non-hospitalized individuals. Second, we only collected serological biomarkers at hospital admission. Accordingly, we do not know if long-lasting inflammation would be present in these patients after acute infection. Third, several inflammatory biomarkers, e.g., cytokines or interleukins, were not analyzed, which may exhibit stronger associations with sensitization-associated symptomatology. Fourth, we used the CSI for inferring the presence of sensitization. Due to the limitations of the CSI as a self-reported outcome for capturing the complexity of altered nociceptive processing [53], studies including objective measures of central nervous system excitability are needed. 

## 5. Conclusions

In conclusion, although patients with sensitization-associated symptoms showed higher lymphocyte count and lower urea levels than those without sensitization-associated symptoms, most of the investigated serological biomarkers at hospital admission were not associated with sensitization-associated pain symptoms. Further, any hospitalization treatment was either associated with the presence of post-COVID pain with or without sensitization-associated symptoms. The presence of other post-COVID symptoms such as fatigue, brain fog, or dyspnea was similar between COVID-19 survivors with and without post-COVID pain with or without sensitization-associated symptoms. 

## Figures and Tables

**Table 1:** Clinical and hospitalization data according to the presence or absence of post-COVID pain.

	No Post-COVID Pain (n = 108)	Post-COVID Pain (n = 75)
Age, mean (SD), years	56.5 (13.5)	57.9 (11.0)
Gender, male/female (%)	57 (52.8%)/51 (47.2%)	37 (49.3%)/38 (50.7%)
Weight, mean (SD), kg.	81.3 (15.9)	80.9 (16.5)
Height, mean (SD), cm.	167.35 (9.25)	167.5 (9.6)
Number of medical comorbidities	1.35 (1.0)	1.3 (1.1)
Medical co-morbidities		
Hypertension	39 (36.1%)	25 (33.3%)
Obesity	38 (35.2%)	19 (25.3%)
Asthma	13 (12.05%)	12 (16.0%)
Cardiovascular diseases	9 (8.3%)	5 (6.7%)
Diabetes *	5 (4.6%)	11 (14.7%)
Chronic obstructive pulmonary disease	4 (3.7%)	1 (1.3%)
Previous pain symptomatology, n (%)	52 (48.15%)	36 (48.0%)
Number of COVID-19 symptoms at hospital admission, mean (SD)	3.4 (0.8)	3.3 (0.9)
Symptoms at hospital admission, n (%)		
Fever	86 (79.6%)	56 (74.7%)
Dyspnea	39 (36.1%)	26 (34.7%)
Myalgias	59 (54.6%)	45 (60.0%)
Cough	38 (35.2%)	30 (40.0%)
Headache	39 (36.1%)	23 (30.7%)
Diarrhea	24 (22.2%)	15 (20.0%)
Anosmia	26 (24.1%)	20 (26.7%)
Ageusia	29 (26.8%)	14 (18.7%)
Throat pain	15 (13.9%)	6 (8.0%)
Vomiting	8 (7.4%)	9 (12.0%)
Dizziness	4 (3.7%)	6 (8.0%)
Stay at the hospital, mean (SD), days	7.9 (6.7)	9.1 (12.1)
Treatments received at hospitalization, n (%)		
Oral corticoids	7 (6.5%)	7 (9.3%)
Inhaled corticoids	10 (9.25%)	13 (17.3%)
Statins	22 (20.4%)	24 (32.0%)
Antiaggregant	10 (9.25%)	6 (8.0%)
Anticoagulants	17 (15.75%)	9 (12.0%)
NSAIDs	19 (17.6%)	17 (22.7%)
Oxygen therapy (no intubation)	36 (33.3%)	34 (45.3%)
Lopinavir/Ritonavir	24 (22.2%)	17 (22.7%)
Hydroxychloroquine	57 (52.8%)	47 (62.7%)
Antibiotics	51 (47.2%)	33 (44.0%)
Tocilizumab	6 (5.55%)	3 (4.0%)
Methylprednisone	9 (8.3%)	7 (9.3%)
Dexamethasone	3 (2.8%)	2 (2.7%)
Heparine	62 (57.4%)	47 (62.7%)

n: number; SD: standard deviation; * Statistically significant differences between groups (*p* < 0.05).

**Table 2:** Laboratory biomarkers according to the presence or absence of post-COVID pain.

	No Post-COVID Pain (n = 108)	Post-COVID Pain (n = 75)
C-Reactive Protein	73.33 (54.38)	81.05 (58.18)
Lactate dehydrogenase (LDH, U/L) *	294.12 (81.91)	266.13 (69.84)
Activated partial thromboplastin time (APTT, seconds)	31.94 (5.34)	31.32 (5.92)
Leucocytes (×10^9^/L)	6.12 (1.72)	6.48 (2.32)
Neutrophils (×10^9^/L) *	4.29 (1.62)	4.77 (1.99)
Lymphocytes (×10^9^/L)	1.3 (0.46)	1.21 (0.55)
Creatine kinase (CK, mg/dL)	0.89 (0.21)	0.96 (0.59)
Alanine transaminase (ALT, U/L)	41.45 (24.05)	37.44 (27.05)
Aspartate transaminase (AST, U/L)	43.15 (20.69)	38.49 (22.92)
Bilirubin (mg/dL)	0.57 (0.24)	0.57 (0.20)
Glucose (mg/mL)	109.73 (30.95)	112.82 (19.92)
Hematocrit (%)	44.88 (3.17)	43.81 (3.92)
Platelets (×109/L)	227.92 (86.28)	227.89 (69.19)
D-dimer (ng/mL)	1022.57 (984.66)	900.07 (813.70)
Albumin (g/dL)	4.53 (0.32)	4.58 (0.28)
Urea (mg/dL)	32.45 (10.84)	33.74 (20.17)
Sodium (mEq/L)	138.76 (3.02)	138.27 (2.48)
Potassium (mmol/L)	4.13 (0.44)	4.12 (0.40)

n: number; SD: standard deviation; * Statistically significant differences between groups (*p* < 0.05).

**Table 3:** Clinical and hospitalization data according to the presence or absence of sensitization-associated symptoms (CSI ≥ 40 points).

	No Post-COVID Pain (n = 108)	CSI < 40 Points (n = 46)	CSI ≥ 40 Points (n = 29)
Age, mean (SD), years	56.5 (13.5)	58.0 (11.0)	57.7 (10.0)
Gender, male/female (%)	57 (52.8%)/51 (47.2%)	17 (58.7%)/19 (41.3%)	10 (34.5%)/19 (65.5%)
Weight, mean (SD), kg.	81.3 (15.9)	81.2 (23.0)	81.25 (23.1)
Height, mean (SD), cm.	167.35 (9.25)	168.35 (10.0)	166.25 (9.1)
Number of medical comorbidities	1.35 (1.0)	1.25 (1.1)	1.30 (1.0)
Medical co-morbidities			
Hypertension	39 (36.1%)	15 (32.6%)	10 (34.5%)
Obesity	38 (35.2%)	12 (26.1%)	7 (24.15%)
Asthma	13 (12.05%)	7 (15.2%)	5 (17.25%)
Cardiovascular diseases	9 (8.3%)	4 (8.7%)	1 (3.5%)
Diabetes	5 (4.6%)	5 (10.9%)	6 (20.7%)
Chronic obstructive pulmonary disease	4 (3.7%)	1 (2.2%)	0 (0.0%)
Previous pain symptomatology, n (%)	52 (48.15%)	19 (41.3%)	17 (58.6%)
Number of COVID-19 symptoms at hospitalization, mean (SD)	3.4 (0.8)	3.2 (0.85)	3.5 (0.9)
Symptoms at hospital admission, n (%)			
Fever	86 (79.6%)	34 (73.9%)	22 (75.9%)
Dyspnea	39 (36.1%)	14 (30.4%)	12 (41.4%)
Myalgias	59 (54.6%)	25 (54.35%)	20 (69.0%)
Cough	38 (35.2%)	18 (39.1%)	12 (41.4%)
Headache	39 (36.1%)	15 (32.6%)	8 (27.6%)
Diarrhea	24 (22.2%)	7 (15.2%)	8 (27.6%)
Anosmia	26 (24.1%)	12 (26.1%)	8 (27.6%)
Ageusia	29 (26.8%)	12 (26.1%)	2 (6.9%)
Throat pain	15 (13.9%)	3 (6.5%)	3 (10.35%)
Vomiting	8 (7.4%)	6 (13.05%)	3 (10.35%)
Dizziness	4 (3.7%)	2 (4.35%)	4 (13.8%)
Stay at the hospital, mean (SD), days	7.9 (6.7)	10.3 (14.5)	7.2 (6.2)
Treatments received at hospitalization, n (%)			
Oral corticoids	7 (6.5%)	4 (8.7%)	3 (10.35%)
Inhaled corticoids	10 (9.25%)	6 (13.05%)	7 (24.15%)
Statins	22 (20.4%)	14 (30.4%)	10 (34.5%)
Antiaggregant	10 (9.25%)	4 (8.7%)	2 (6.9%)
Anticoagulants	17 (15.75%)	7 (15.2%)	2 (6.9%)
NSAIDs	19 (17.6%)	12 (26.1%)	5 (17.25%)
Oxygen Therapy (no intubation)	36 (33.3%)	23 (50.0%)	11 (37.9%)
Lopinavir/Ritonavir	24 (22.2%)	14 (30.45%)	3 (10.35%)
Hydroxychloroquine	57 (52.8%)	28 (60.9%)	19 (65.5%)
Antibiotics	51 (47.2%)	23 (50.0%)	10 (34.5%)
Tocilizumab	6 (5.55%)	2 (4.35%)	1 (3.45%)
Methylprednisone	9 (8.3%)	5 (10.9%)	2 (6.9%)
Dexamethasone	3 (2.8%)	2 (4.35%)	0 (0.0%)
Heparin	62 (57.4%)	31 (67.4%)	16 (55.2%)

n: number; SD: standard deviation;

**Table 4:** Laboratory biomarkers according to the presence or absence of sensitization-associated symptoms (CSI ≥ 40 points).

	No Post-COVID Pain (n = 108)	CSI < 40 Points (n = 46)	CSI ≥ 40 Points (n = 29)
C-Reactive Protein	73.33 (54.38)	83.55 (56.17)	77.09 (62.04)
Lactate dehydrogenase (LDH, U/L) *	294.12 (81.91)	269.31 (73.84)	261.08 (63.91)
Activated partial thromboplastin time (APTT, seconds)	31.94 (5.34)	32.12 (6.55)	30.06 (4.58)
Leucocytes (×10^9^/L) *	6.12 (1.72)	6.43 (2.03)	6.56 (2.74)
Neutrophils (×10^9^/L)	4.29 (1.62)	4.85 (1.82)	4.64 (2.25)
Lymphocytes (×10^9^/L) #	1.3 (0.46)	1.1 (0.51)	1.4 (0.57)
Creatine kinase (CK, mg/dL)	0.89 (0.21)	1.01 (0.73)	0.87 (0.24)
Alanine transaminase (ALT, U/L)	41.45 (24.05)	39.47 (29.08)	34.23 (23.61)
Aspartate transaminase (AST, U/L)	43.15 (20.69)	40.04 (23.5)	36.02 (22.14)
Bilirubin (mg/dL)	0.57 (0.24)	0.62 (0.2)	0.50 (0.18)
Glucose (mg/mL)	109.73 (30.95)	111.01 (16.89)	115.7 (24.01)
Hematocrit (%)	44.88 (3.17)	43.85 (4.18)	43.74 (3.54)
Platelets (×10^9^/L)	227.92 (86.28)	227.0 (70.5)	229.31 (68.28)
D-dimer (ng/mL)	1022.57 (984.66)	801.97 (627.91)	1055.68 (1036.97)
Albumin (g/dL)	4.53 (0.32)	4.53 (0.24)	4.65 (0.32)
Urea (mg/dL) #	32.45 (10.84)	37.3 (23.18)	28.09 (12.54)
Sodium (mEq/L)	138.76 (3.02)	138.17 (2.81)	138.43 (1.87)
Potassium (mmol/L)	4.13 (0.44)	83.55 (56.17)	77.09 (62.04)

n: number; SD: Standard Deviation; * Statistically significant differences between patients with and without pain (*p* < 0.05); # Statistically significant differences between patients with post-COVID pain with and without sensitization-associated symptomatology (*p* < 0.05).

**Table 5:** Other post-COVID symptomatology according to the presence or absence of sensitization-associated symptoms (CSI ≥ 40 points).

	No Post-COVID Pain (n = 108)	CSI < 40 Points (n = 46)	CSI ≥ 40 Points (n = 29)
Fatigue, n (%)	60 (55.5%)	27 (58.7%)	20 (69.0%)
Dyspnea at exertion, n (%)	69 (63.9%)	30 (65.2%)	19 (65.5%)
Brain fog, n (%)	16 (14.8%)	4 (8.7%)	6 (20.7%)
HADS-A (0–21), mean (SD)	3.2 (3.6)	3.3 (3.7)	4.4 (4.5)
Anxiety Symptoms (HADS-A ≥8), n (%)	13 (12.05%)	6 (13.05%)	5 (17.25%)
HADS-D (0–21), mean (SD) *	2.25 (3.1)	2.25 (2.6)	4.0 (4.2)
Depressive symptoms (HADS-A ≥8), n (%) *	7 (6.5%)	2 (4.35%)	6 (20.7%)
PSQI (0–21), mean (SD)	6.7 (3.9)	5.35 (3.8)	6.6 (3.5)
Poor Sleep (PSQI ≥8 points), n (%)	44 (40.75%)	9 (19.57%)	11 (37.9)
CSI (0–100), mean (SD)	---	22.9 (10.1)	54.9 (9.1)
PCS (0–52), mean (SD)	---	5.5 (8.1)	10.8 (7.3)
Pain intensity (NPRS, 0–10), mean (SD)	---	5.6 (1.7)	5.5 (1.8)

n: number; SD: standard deviation; * Statistically significant differences between groups (*p* < 0.05); HADS: Hospital Anxiety and Depression Scale; PSQI: Pittsburgh Sleep Quality Index; CSI: Central Sensitization Inventory; PCS: Pain Catastrophizing Scale; NPRS: Numerical Pain Rate Scale.

## Data Availability

All data relevant to the study are included in the article.

## Supplementary Materials

The following supporting information can be downloaded at: https://www.mdpi.com/article/10.3390/pathogens12101235/s1.
